# Fucosylated oligosaccharides in mother’s milk alleviate the effects of caesarean birth on infant gut microbiota

**DOI:** 10.1038/s41598-018-32037-6

**Published:** 2018-09-13

**Authors:** Katri Korpela, Anne Salonen, Brandon Hickman, Clemens Kunz, Norbert Sprenger, Kaarina Kukkonen, Erkki Savilahti, Mikael Kuitunen, Willem M. de Vos

**Affiliations:** 1 Immunobiology Research Programme, Department of Bacteriology and Immunology, University of Helsinki, Helsinki, Finland; 2 European Molecular Laboratory, Heidelberg, Germany; 3 Institute of Nutritional Sciences, Justus-Liebig University Giessen, 35392 Giessen, Germany; 4 Nestlé Research Center, Nestec S.A., Vers-Chez-Les-Blanc, 26, Lausanne, 1000 Switzerland; 5 Skin and Allergy Hospital, Department of Paediatrics, Helsinki University Central Hospital, Helsinki, Finland; 6 Children’s Hospital, University of Helsinki and Helsinki University Central Hospital, Helsinki, Finland; 7 Laboratory of Microbiology, Wageningen University, Wageningen, The Netherlands

## Abstract

One of the most abundant components in human milk is formed by oligosaccharides, which are poorly digested by the infant. The oligosaccharide composition of breast milk varies between mothers, and is dependent on maternal secretor (FUT2) genotype. Secretor mothers produce milk containing α1-2 fucosylated human milk oligosaccharides, which are absent in the milk of non-secretor mothers. Several strains of bacteria in the infant gut have the capacity to utilise human milk oligosaccharides (HMOs). Here we investigate the differences in infant gut microbiota composition between secretor (N = 76) and non-secretor (N = 15) mothers, taking into account birth mode. In the vaginally born infants, maternal secretor status was not associated with microbiota composition. In the caesarean-born, however, many of the caesarean-associated microbiota patterns were more pronounced among the infants of non-secretor mothers compared to those of secretor mothers. Particularly bifidobacteria were strongly depleted and enterococci increased among the caesarean-born infants of non-secretor mothers. Furthermore, *Akkermansia* was increased in the section-born infants of secretor mothers, supporting the suggestion that this organism may degrade HMOs. The results indicate that maternal secretor status may be particularly influential in infants with compromised microbiota development, and that these infants could benefit from corrective supplementation.

## Introduction

Infants are adapted to obtaining all of their nutrition from human milk during the first months of life. In addition to nutrients for the infant, breast milk contains a diverse mixture of complex oligosaccharides, termed human milk oligosaccharides (HMOs), at an abundance of approximately 10 g/l^1^. These oligosaccharides are poorly digested by the infant, but are favoured growth substrates for intestinal bacteria that have the appropriate enzymatic degradation capacity. The oligosaccharide composition and abundance in breast milk is dependent on maternal genetics, particularly the FUT2 gene, which encodes an enzyme responsible for the addition of fucose at the α1-2 position on a backbone of abundant glycans containing galactose^1^. The breast milk of mothers with a functional FUT2 allele, the so-called secretors, contains a large amount of α1-2 fucosylated HMOs, most abundantly 2′fucosyllactose (2′FL), and in lesser amounts lactodifucotetraose (LDFT), lacto-N-difucohexaose I (LNDFH I) and lacto-*N*-fucopentaose I (LNFP I)^1,2^. The breast milk of non-secretor mothers lacks or has only traces of these α1-2 fucosylated oligosaccharides, thus containing a lower total amount of HMOs^1,2^, although this lack may be partly compensated by higher abundances of lacto-N-tetraose (LNT), LNFP II, and III and LNDFH II^1^. The abundance of 2′FL in breast milk has been shown to be a reliable indicator of secretor status^2^.

Maternal secretor phenotype has been recently linked with reduced risk of atopic dermatitis in a cohort of caesarean-born infants^3^, and individual HMOs were related to reduced risk of cow’s milk allergy^4^. Although HMOs are reported to have immunomodulatory effects, these are mainly restricted to sialylated HMOs^5,6^, which are not dependent on maternal secretor status. Although some fucosylated HMOs like 2′ FL can bind the dendritic cell receptor DC-SIGN^7^ and modulate inflammation via CD14^8^, it is not known whether this leads to direct allergy-related immunological effects.

One likely manner in which maternal secretor status influences the infant’s immune system development is via the gut microbiota. The α1-2 fucosylated oligosaccharides are degraded by enzymes of the glycosyl hydrolase family 95, possessed by strains of bifidobacteria commonly observed in infants (strains of *B. longum infants, B. bifidum, B. breve)* as well as *Bacteroides* spp.^9–13^. The abundance of these bacteria is associated with birth mode, being commonly reduced in caesarean-born infants^14^. We therefore hypothesise that the impact of maternal secretor status on infant microbiota may depend on birth mode. Here we investigate the differences in infant gut microbiota composition between secretor and non-secretor mothers, taking into account birth mode.

## Results

**Figure 1:**
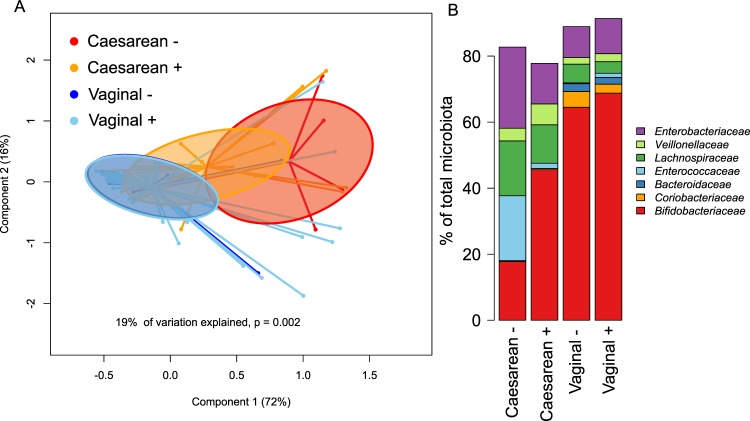
Overall infant microbiota composition by birth mode and maternal secretor status. (**A**) PCoA using Bray-Curtis dissimilarities. (**B**) Average relative abundance of the most abundant families per group.

We analysed the association between microbiota composition in infants and the presence of 2′FL in maternal breast milk (secretors), stratifying the infants by birth mode. The stratification of the samples into groups based on birth mode and the presence of 2′FL in maternal breast milk explained 19% the inter-individual variation in phylum-level microbiota composition (Fig. 1A). The maternal secretor phenotype was not associated with the overall microbiota composition among the vaginally born infants (Fig. 1A; permutational multivariate ANOVA, p = 0.98). Among the caesarean born infants, however, the maternal secretor phenotype was associated with an overall shift in the total microbiota composition, which was close to statistical significance (Fig. 1A, p = 0.07). The caesarean-born infants of secretor mothers had a more modest deviation in microbiota composition, compared to those of non-secretor mothers (Fig. 1A,B).

**Figure 2:**
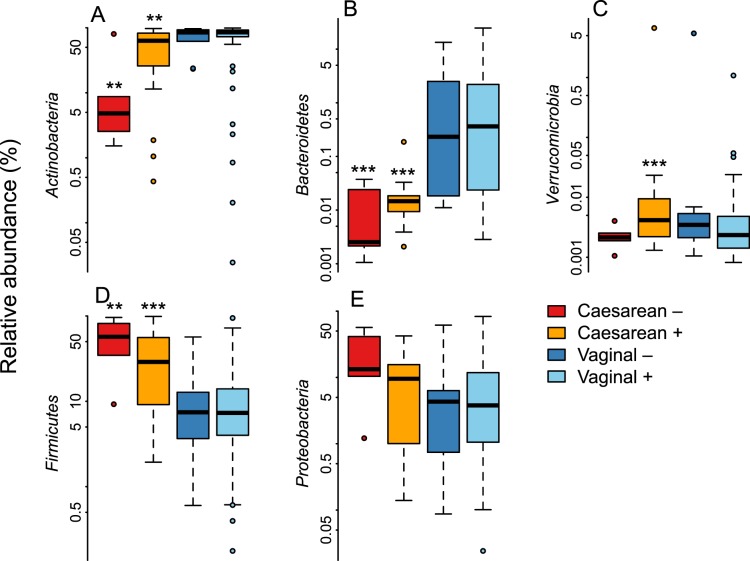
Relative abundance of the bacterial phyla by birth mode and maternal secretor status. The asterisks indicate the significance of the difference to the vaginally born infants of secretor mothers: ^**^p < 0.01, ^***^p < 0.001.

**Figure 3:**
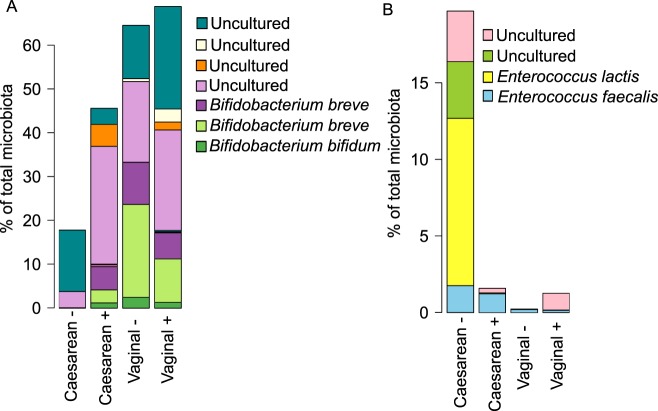
Average relative abundance of (**A**) *Bifidobacterium* species and (**B**) *Enterococcus* species by birth mode and maternal secretor status. “Uncultured” means that the species has not been cultured and characterised, and therefore has not been named.

Among the vaginally born infants, there were no significant differences between infants of secretor and non-secretor mothers in the relative abundance of bacterial taxa, after adjustment for full or partial breastfeeding and at the FDR-corrected p-value level 0.1 (Supplementary Table 1). All caesarean-born infants had significant reductions in the relative abundance of Bacteroidetes (non-secretors, 500-fold reduced, p < 0.0001; secretors, 200-fold reduced, p < 0.0001, mainly genera *Bacteroides* and *Parabacteroides*) and an increase of Firmicutes (non-secretors, 3.8-fold, p = 0.002; secretors 2.6-fold, p = 0.0002, mainly *Bacilli*) in their gut microbiota, compared to the vaginally born infants of secretor mothers. The magnitude of these effects was higher in the infants of non-secretor mothers (Supplementary Table 1, Fig. 2), who also showed a significant reduction in Actinobacteria (3.7-fold, p = 0.001, mainly *Bifidobacterium*) and a 13-fold increase in *Enterococcus* (p = 0.003). The reduction in bifidobacteria was largely due to the absence of *Bifidobacterium bifidum* and *B. breve* (Fig. 3A), while the increase in enterococci was attributable to a bloom of *Enterococcus lactis* and two uncultured *Enterococcus* species; *Enterococcus faecium* levels were comparable (Fig. 3B). In addition, there was a non-significant trend towards higher Proteobacteria abundance in the caesarean-born infants of non-secretor mothers (Fig. 2, Supplementary Table 1), and the caesarean-born infants of secretor mothers had significantly increased relative abundance (approximately ten-fold) of Verrucomicrobia (*Akkermansia muciniphila*, Fig. 2, Supplementary Table 1).

**Figure 4:**
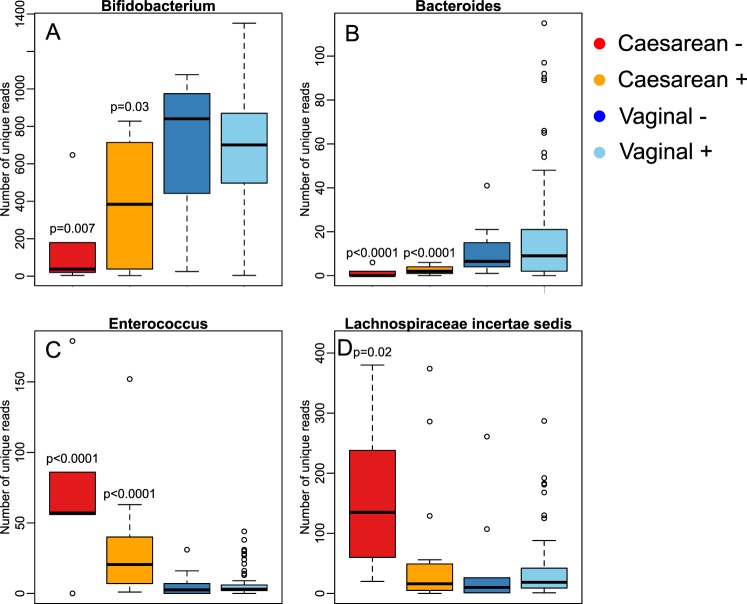
Sequence-level diversity (richness) within selected genera. The p-values represent the significance of the difference to the vaginally born infants of secretor mothers (“Vaginal +”), from negative binomial regression.

In addition to the differences in relative abundance, we observed differences in the sequence-level diversity of several genera, indicating variation in the diversity of species and strains. The diversity (number of unique sequences assigned to the particular genus) of bifidobacteria and bacteroides was significantly reduced in the caesarean-born infants, especially clearly in the infants of non-secretor mothers (Fig. 4A,B), while the diversity of enterococci and *Lachnospiraceae incertae sedis* showed the opposite pattern (Fig. 4C,D).

## Discussion

Our results indicate that infants of non-secretor mothers are particularly vulnerable to the effects of caesarean section on the early microbiota development. The combination of caesarean birth and lack of milk 2′FL appear to profoundly alter the infant’s microbiota.

A few previous studies have investigated the association between maternal secretor status and infant microbiota. Low abundance of bifidobacteria has been reported in infants of non-secretor mothers^15^, corresponding to our observations among the caesarean-born infants, but these associations are not universal. Wang *et al*. report a negative correlation between 2′FL and LDFT in breast milk and the relative abundance of bifidobacteria in infant faeces, but a positive correlation with *Bacteroides*^16^. This suggests that the principal degraders of fucosylated HMOs may vary between cohorts, depending on the composition of the microbiota. In our cohort, maternal secretor status had no impact on the microbiota composition among the vaginally born infants, indicating that maternal secretor status may be an important factor mainly among infants with otherwise compromised microbiota development. Curiously, the overall abundance of bifidobacteria in the cohort of Lewis *et al*. (2015) was similar to that of the caesarean-born infants in our cohort. The HMO degradation capacity of bifidobacteria is not restricted to the α1-2 fucosylated HMOs^17,18^. Perhaps a high diversity of bifidobacteria ensures that the community is able to adapt to the available HMOs, reducing their dependence on specific HMO types.

In the caesarean-born infants, the only taxon appearing to benefit from the lack of α1-2 fucosylated HMOs was *Enterococcus* (member of *Lactobacillales*), particularly *Enterococcus lactis*. *E. lactis* is a non-pathogenic, originally dairy-derived bacterium with strong antimicrobial activity against other Gram-positive bacteria, such as other enterococci and clostridia^19,20^. It is possible that this organism, which utilises lactose and galactose^19^, can opportunistically take advantage of the available niche in the infant gut when the abundance of normally dominant bacteria is low and successfully outcompete other similar bacteria. Instead of enterococci, Lewis *et al*. discovered an increased abundance of streptococci, also belonging to the class *Lactobacillales*, in the infants of non-secretor mothers^15^, while Underwood *et al*. report a reduced abundance of *Lactobacillales* in premature infants of non-secretor mothers^21^. Most lactobacilli do not degrade complex HMOs^22^, so the latter result is likely due to indirect effects of the HMOs on other bacteria.

In infants of non-secretor mothers there seems to be a trend towards increased abundance of enterobacteria^15,21^, which we also observed in the caesarean born infants. Many members of enterobacteria are inflammatory, some even pathogenic. Fucosylated oligosaccharides are decoy receptors for many pathogenic bacteria, including members of enterobacteria; hence, they have a potential to reduce their adhesion to the gut, thus protecting the infant^23–25^. In addition, fucosylated HMOs bind enterotoxin produced by *E. coli* and inhibit its effects on intestinal cells^26^, and reduce LPS-induced inflammation in gut epithelium^8^. Fucosylated HMOs may thus reduce the colonisation ability of enterobacteria as well as their inflammatory effects in the infant gut, particularly among prematurely born infants.

A novel finding in our study was the increased abundance of *Akkermansia* in the caesarean-born infants of secretor mothers. *Akkermansia muciniphila* is able to degrade HMOs^27^, and thus may benefit from the presence of 2′FL in the situation in caesarean-born infants where normally abundant HMO-degrading members of the community, bifidobacteria and bacteroides, are depleted. *Akkermansia* strengthens the gut barrier and likely contributes positively to infant gut health^28^.

Low total abundance of bifidobacteria, and low abundance of typical infant-type bifidobacteria, in infancy has been associated with allergic disease^29–31^, which may explain the observed associations between maternal secretor status and infant allergy risk^3,4^. Several bifidobacterial strains have immunomodulatory effects^32–36^. Consequently, supplementation with bifidobacteria has been shown to reduce the incidence of allergic diseases in infants^37^.

Our results show that caesarean birth may be especially detrimental to the microbiota of infants fed by non-secretor mothers, potentially leading to increased risk of allergic diseases^3,4^. Screening for FUT2 genotype or 2′FL in the breast milk of mothers giving birth by caesarean section would allow these at-risk infants to be identified and treated. These infants would most likely benefit from specific interventions, such as supplementation with bifidobacteria, bifidogenic 2′fucosylated HMOs like 2′FL, or other compounds that could be effective in reducing their allergy incidence.

## Methods

We collected breast milk samples on postpartum day 3 and infant faecal samples at age 3 months during a probiotic intervention trial^38^. The study was approved by the medical ethical board of Helsinki and Uusimaa Hospital District, Finland and was conducted according to the Declaration of Helsinki. Parents provided written informed consent. For this study, we selected a subset of the original cohort with the following criteria: both samples available, infant breastfed (exclusively or partially) at the time of faecal sample collection, placebo treatment during the trial (no probiotic given to infant or mother), and no antibiotics given to the infant prior to faecal sample collection. In total we included 76 infants of secretor mothers (58 vaginally born, 62% fully breastfed, and 18 caesarean born, 33% fully breastfed) and 15 infants of non-secretor mothers (10 vaginally born, 70% fully breastfed, and 5 caesarean born, 67% fully breastfed).

Breast milk samples were assigned to a group (i) with FUT2 dependent milk oligosaccharides (FUT2 positive) and (ii) without FUT2 dependent milk oligosaccharides (FUT2 negative) using 2 independent methods, as described earlier^3,39^. Briefly, skimmed breast milk samples were analysed using MALDI-TOF (matrix assisted laser desorption/ionization - time of flight) mass spectrometry (MS) profiling and liquid chromatography to quantify 2′fucosyllactose (2′FL) and by high performance anion exchange chromatography (HPAEC) with a CarboPac PA1 analytical column coupled to a pulsed amperometry detector (ICS3000, Thermo Fischer Dionex, Sunnyvale, USA). The distribution of 2′FL values was close to normal. The mean ± sd level of 2′FL in the milk of secretor mothers was 5664 ± 2048 mg/l (vaginal birth) and 3810 ± 1183 mg/l (Caesarean birth). The amount was significantly lower in the Caesarean group (ANOVA, p = 0.0003). The mean amount in the milk of non-secretor mothers was below 50 mg/l.

Faecal samples were analysed for microbiota composition by 16S rRNA amplicon sequencing. DNA was extracted from the faecal samples using a repeated bead-beating protocol^40^. The library preparation was performed essentially according to the protocol by Illumina, except that the 16S rRNA gene amplification and barcoding was performed in a single reaction. The PCR reaction comprised 1 ng/μl template, 1X Phusion® Master Mix (ThermoFisher, catalog number: F-531L), 0.25 μM V3-V4 locus specific primers and 0.375 μM TruSeq dual-index primers. The PCR was run under the following settings: 98 °C for 30 s, 27 cycles of 98 °C for 10 s, 62 °C for 30 s, 72 °C for 15 s and finally 10 min at 72 °C, where after the samples were stored at 4 °C. The PCR clean-up was performed with AMPure XP beads (Beckman Coulter, Copenhagen, Denmark) and confirmation of the correct amplicon size (ca. ~640 base pairs) was performed on a Bioanalyzer DNA 1000 chip (Agilent Technology, Santa Clara, CA, USA). The pooled libraries were sequenced with an Illumina MiSeq or HiSeq2500 in Rapid Run mode. The median number of reads obtained per sample was 46 260 (range 4260-137 400). The samples were randomly assigned to the different sequencing platforms. To make sure the results don’t suffer from technical bias, we sequenced three artificial communities of known composition on both platforms and compared the results (Supplementary Fig. 1). No systematic difference was observed between the different sequencing platforms. The sequencing platform did not explain a significant amount of variation in the real data (p = 0.3, multivariate permutational anova).

Statistical analysis was conducted in R, using the package mare^41^, with tools from packages vegan^42^, MASS^43^, and nlme^44^. Although paired-end sequencing was conducted, we only used the forward reads truncated to 150 bases, as we have observed using artificial communities of known composition that longer reads provide unreliable results^45^. Taxonomic annotation was performed using USEARCH^46^ by mapping the reads to the SILVA 16S rRNA reference database version 115^47^, restricted to gut-associated taxa (available through the R package mare^41^). We did not rarefy the data, but instead used the number of reads per sample as the offset in the models. We adjusted for full or partial breastfeeding in the models. Due to multiple testing, we considered FDR-corrected p < 0.1 as significant, but report uncorrected p-values in the text for clarity. Both corrected and uncorrected p-values are presented in Supplementary Table 1. For reliable results, it is extremely important that the data follow the underlying assumptions of the statistical test (particularly independence and normal distribution of residuals). The R-package mare addresses this issue by iteratively finding the most suitable model for each taxon. For this reason, the statistical test is not the same for all taxa, as the distribution of the data varies between taxa. Very commonly, there are patterns in the residuals that must be adjusted for. In these cases, generalised least squares models are used that allow for the residual variation to be modelled separately.

## Electronic supplementary material

Supplementary Figure 1

Supplementary Table 1

## Data Availability

The DNA sequencing data are available at ENA (http://www.ebi.ac.uk/ena) under the accession number PRJEB27325.
