# Supplementary material for: Fucosylated oligosaccharides in mother’s milk alleviate the effects of caesarean birth on infant gut microbiota
Source: Sci Rep. 2018 Sep 13;8:13757. doi: 10.1038/s41598-018-32037-6 (PMC6137148; doi:10.1038/s41598-018-32037-6)
Supplement: Supplementary file 1 — Supplementary Figure 1 [file 41598_2018_32037_MOESM1_ESM.docx]

Supplementary Information

Fucosylated oligosaccharides in mother’s milk alleviate the effects of caesarean birth on infant gut microbiota

Katri Korpela^a,b^ *, Anne Salonen^a^ , Brandon Hickman^a^, Clemens Kunz^c^, Norbert Sprenger^d^, Kaarina Kukkonen^e^ , Erkki Savilahti^f^ , Mikael Kuitunen^f^ , Willem M. de Vos^a,g^

*Corresponding author, katri.korpela@helsinki.fi

Affiliations: ^a^Immunobiology Research Programme, Department of Bacteriology and Immunology, University of Helsinki, Helsinki, Finland; ^b^European Molecular Laboratory, Heidelberg, Germany; ^c^Institute of Nutritional Sciences, Justus-Liebig University Giessen, 35392 Giessen, Germany ; ^d^Nestlé Research Center, Nestec S.A., Vers-Chez-Les-Blanc, 26 Lausanne 1000, Switzerland ; ^e^Skin and Allergy Hospital, Department of Paediatrics, Helsinki University Central Hospital, Helsinki, Finland; ^f^Children’s Hospital, University of Helsinki and Helsinki University Central Hospital, Helsinki, Finland; ^g^Laboratory of Microbiology, Wageningen University, Wageningen, the Netherlands

Supplementary Figure 1. Principal coordinates analysis of three mock communities of known composition, sequenced on different platforms. The samples were sequenced on HiSeq once and on MiSeq four times. The MiSeq samples were prepared using a 1-step and 2-step PCR protocol, both replicated twice.
